# Endocannabinoids in Body Weight Control

**DOI:** 10.3390/ph11020055

**Published:** 2018-05-30

**Authors:** Henrike Horn, Beatrice Böhme, Laura Dietrich, Marco Koch

**Affiliations:** Institute of Anatomy, Medical Faculty, University of Leipzig, 04103 Leipzig, Germany; Henrike.Horn@medizin.uni-leipzig.de (H.H.); Beatrice.Schuetzelt@medizin.uni-leipzig.de (B.B.); lauri.dietrich@yahoo.de (L.D.)

**Keywords:** body weight, obesity, anorexia, cancer cachexia, endocannabinoids, cannabinoid type 1 receptor, CB1, allosteric CB1 ligands

## Abstract

Maintenance of body weight is fundamental to maintain one’s health and to promote longevity. Nevertheless, it appears that the global obesity epidemic is still constantly increasing. Endocannabinoids (eCBs) are lipid messengers that are involved in overall body weight control by interfering with manifold central and peripheral regulatory circuits that orchestrate energy homeostasis. Initially, blocking of eCB signaling by first generation cannabinoid type 1 receptor (CB1) inverse agonists such as rimonabant revealed body weight-reducing effects in laboratory animals and men. Unfortunately, rimonabant also induced severe psychiatric side effects. At this point, it became clear that future cannabinoid research has to decipher more precisely the underlying central and peripheral mechanisms behind eCB-driven control of feeding behavior and whole body energy metabolism. Here, we will summarize the most recent advances in understanding how central eCBs interfere with circuits in the brain that control food intake and energy expenditure. Next, we will focus on how peripheral eCBs affect food digestion, nutrient transformation and energy expenditure by interfering with signaling cascades in the gastrointestinal tract, liver, pancreas, fat depots and endocrine glands. To finally outline the safe future potential of cannabinoids as medicines, our overall goal is to address the molecular, cellular and pharmacological logic behind central and peripheral eCB-mediated body weight control, and to figure out how these precise mechanistic insights are currently transferred into the development of next generation cannabinoid medicines displaying clearly improved safety profiles, such as significantly reduced side effects.

## 1. Introduction

It has evolved in human and most other species that the body weight remains relatively constant for most of the lifetime. In other words, an individual able to balance the body weight long-term was successful and survived, most likely because body weight stability would ultimately have guaranteed a sustained energy supply [1]. Even before becoming adults, species-specific interrelations exist between body weight gain and longitudinal growth during pre-and postnatal development [2]. Thus, when occurring in physiological ranges, body weight development and maintenance are fundamental to maintain health and to promote longevity, while underweight, overweight, and specifically obesity in childhood, adolescence and adulthood are associated with adverse health consequences throughout the life course [3].

At first glance, the present body weight calculation of an individual mostly reflects the latest intake, storage and expenditure of energy. Indeed, the control of energy metabolism strongly accounts for the individual’s body weight [4]. In this, various regulatory circuits in the central nervous system (CNS) and the periphery orchestrate the maintenance of energy homeostasis. First of all, energy intake in terms of food ingestion is supervised in the CNS. Here, environmental and metabolic information is received, integrated and finally transformed into generation of physiological behaviors such as food foraging and energy expenditure in order to provide the energy required for differentiation, growth, regeneration and maintenance of all cells, tissues and organs of the body [5].

### 1.1. Overeating and Obesity—What Is the Evolutionary Benefit of Fat Storage?

Assuming that the presence of sufficient food represented a selective pressure in evolution, one beneficial adaptation apparently was the opportunity to long-term store excess of energy in the body’s fat depots [6]. Accordingly, so-called “pro-feeding” regulatory circuitries, in which energy consumption dominates energy expenditure, evolved as an indispensable prerequisite allowing for the storage of energy [7]. Besides food scarcity, also other selective pressures would have been accounted for these beneficial adaptations. These include the avoidance of predators and that the immune system was able to use the internal energy resources to overcome debilitating diseases such as infections [8]. However, since food is sufficiently available in today’s world, the aforementioned pro-feeding behavioral outcome in which overeating is favored over fasting, in combination with continuous reduction in physical activity, has led to a global obesity epidemic within the last century [9]. Since obesity is a major risk factor for severe secondary diseases such as type 2 diabetes, cardiovascular and neurological diseases and certain kinds of cancer, basic research and clinical studies are dedicating a lot of efforts in order to develop weight loss strategies and obesity therapeutics.

### 1.2. The Endocannabinoid System—A Reliable Partner in Body Weight Control?

One important prerequisite to develop therapeutic interventions such as anti-obesity drugs is the discovery and better understanding of the cellular and molecular elements of the pro-feeding circuitries in our body. Interestingly, one class of endogenous signaling molecules, the so-called endocannabinoids (eCBs), was identified as a highly conserved group of molecules that significantly contributes to metabolic control. Compared to the millennia-old use of cannabis, which is one of the oldest crops cultivated by humankind, the understanding of the basic mechanisms underlying eCB action is a very recent achievement. First uses of hemp for its fibers and as a food source in China can be traced back as far as 6000 years, a first documentation of using cannabis as a medical remedy putatively dates back more than 4000 years [10]. Recreational and medical cannabis use throughout history can be found in many cultures and all over the world [11,12]. However, starting only slightly more than 50 years ago, with the isolation and structural elucidation of cannabidiol [13] and tetrahydrocannabinol (THC) [14,15], the cannabinoid signaling system causing the well-known and medically appreciated effects of Cannabis sativa was revealed piecemeal by the scientific community [16]. After decades of pioneering the uncovering of the cannabinoid system, Mechoulam stated that “[C]annabinoids represent a medicinal treasure trove which waits to be discovered.” [17]. In this article we will review the involvement of the endocannabinoid system (ECS) in body weight control both centrally and peripherally, arguing that cannabinoids and congeners represent compounds and targets of promising potential for the treatment of eating disorders and metabolic disturbances.

### 1.3. Biochemistry of the Endocannabinoid System—An Outline

Evidence that cannabinoids act through a receptor in the brain was found in the late eighties of the last century [18]. The two cannabinoid type 1 (CB1) and 2 (CB2) receptors are G protein-coupled receptors (GPCRs) expressed in virtually all tissues of the body. While CB1 is more abundant in the CNS, CB2 is the predominant cannabinoid receptor in the periphery, especially in cells of the immune system [19,20]. Four years after the discovery of the receptors, the first endogenous ligand for cannabinoid receptors, *N*-arachidonoylethanolamine (anandamide, AEA), was identified [21] and later supplemented by 2-arachidonylglycerol (2-AG) [22], marking the breakthrough that initiated a whole new field of research investigating the ECS [23].

**Figure 1:**
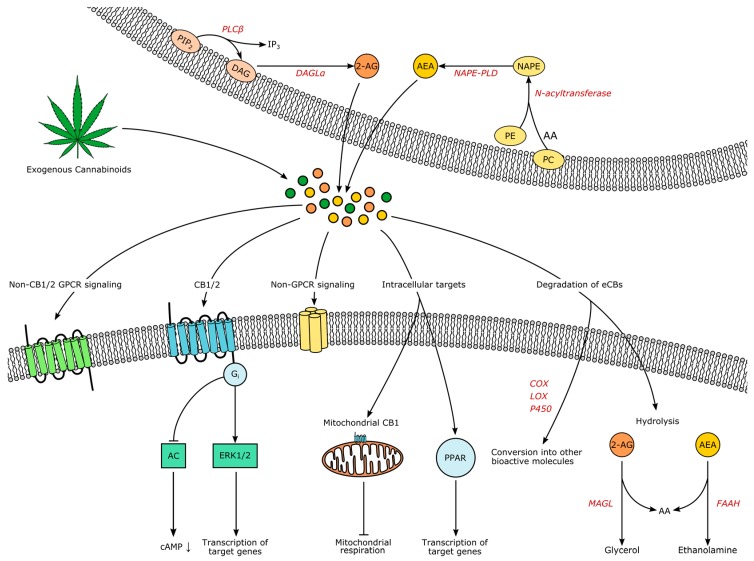
The endocannabinoids (eCBs) AEA and 2-AG are produced on demand from lipid precursors and released to the extracellular space. Endogenous and exogenous cannabinoids act through the same signaling systems: Binding to Gi-coupled receptors CB1 or CB2 modulates intracellular cascades and leads for example to the inhibition of adenylyl cyclase (AC) or the regulation of transcription through extracellular signal-regulated kinases (ERKs). Alternative receptors are non-CB1/2 GPCRs, non-GPCRs like TRPV1 and, intracellularly, mitochondrial CB1 (mtCB1) and peroxisome proliferator-activated receptors (PPARs). Signaling is terminated through hydrolysis, but eCBs might also serve as substrates for cyclooxygenases (COXs), lipoxygenases (LOXs) or cytochromes P450 (P450), yielding additional bioactive compounds. Note that all illustrated processes do not have to take place in distinct cells as autocrine eCB signaling has been shown as well. Abbreviations: PIP2 phosphatidylinositol 4,5-bisphosphate, IP3 inositol-1,4,5-trisphosphat, DAG Diacylglycerol, PLC phospholipase C, DAGL diacylglycerol lipase, 2-AG 2-arachidonylglycerol, PC phosphatidylcholine, PE phosphatidylethanolamine, AA arachidonic acid, NAPE *N*-arachidonoyl phosphatidylethanolamine, NAPE-PLD NAPE-specific phospholipase D, MAGL monoacylglycerol lipase, FAAH fatty acid amide hydrolase.

Many endogenous and synthetic compounds that are part of or interfere with the ECS have been identified and developed (reviewed in [16,24,25]), offering insights into the mechanisms of eCB signaling as well as bearing potential for future treatments. Even though more eCBs have been found, AEA and 2-AG remain the best-characterized representatives. Synthesis, signaling and degradation of these two compounds are visualized in Figure 1. 

Both lipophilic molecules are synthesized from membrane phospholipids on demand upon intracellular Ca^2+^-elevation, following concomitant activation of receptors [26,27]. The precursor of AEA is *N*-arachidonoyl phosphatidylethanolamine (NAPE), formed by the transfer of arachidonic acid from phosphadityl-choline to phosphatidylethanolamine by an enzyme yet to be characterized, followed by formation of AEA by NAPE-specific phospholipase D (NAPE-PLD) [25]. However, additional alternative pathways have been suggested [28]. AEA and 2-AG do not seem to be complementary: decreased brain levels of AEA after global NAPE-PLD-knockout did not increase 2-AG in the CNS, except for the brainstem [29]. Leishman et al. demonstrated that knockout of NAPE-PLD causes extensive lipidome changes beyond *N*-acetylethanolamines, for example the elevation of prostaglandins, providing strong evidence for the underappreciated complexity of the ECS and its relationship to other lipid messenger systems [29]. 

For the synthesis of 2-AG, phoshpholipase C β catalyzes the hydrolysis of phosphatidylinositol 4,5-bisphosphate to diacylglycerol, which serves as the substrate for diacylglycerol lipases (DAGL) α and β [25]. Signaling of AEA is terminated through uptake and intracellular degradation by fatty acid amide hydrolase (FAAH), while 2-AG is degraded by monoacylglycerol lipase (MAGL) [25]. Compared to classical neurotransmitters and -modulators, eCB transport mechanisms are less well understood. The existence of eCB transporters controlling release and uptake is under debate [30,31,32,33]. Due to their hydrophobic nature, eCBs are dependent on binding proteins in aqueous environments, such as albumin [34]. However, how eCBs travel into, through and out of the extracellular space remains enigmatic. As all processes influencing the temporospatial characteristics of eCB signaling constitute potential drug targets, research elucidating these processes is imperatively needed. 

In the classical view, activation of CB1 and CB2 leads to G_i_-mediated inhibition of adenylyl cyclase and a subsequent closure of calcium and opening of potassium channels, underlying the proposed retrograde mode of eCB signaling in the CNS: activated postsynaptic neurons release eCBs, which lower presynaptic intracellular Ca^2+^ and therefore decrease the probability of transmitter release. Depending on the nature of the presynaptic cell, this process is known as ‘depolarization-induced suppression of excitation’ (DSE) or ‘inhibition’ (DSI) [35]. However, more signaling pathways have been discovered, for example activation of the mitogen-activated protein kinase (MAPK) cascades that control cell proliferation, differentiation and death [36]. The eCB signaling has the potential to alter transcription through the MAPK pathway synergistically with other neuropeptides [37]. Also, the universal coupling to G_i_ did not remain unchallenged [36,38], non-retrograde pathways have been identified, such as autocrine inhibition [39], and non-CB1/2 signaling has been discovered, for example through the cation channel transient receptor potential vanilloid 1 (TRPV1), through peroxisome proliferator-activated receptors (PPAR) and potentially through additional GPCRs like GPR55 [40]. Interestingly, CB1 is also located in mitochondria of neural cells (termed “mtCB1”), and the activation of mtCB1 decreases mitochondrial activity and respiration and therefore affects neuronal activity [41]. Additionally, it is now clear that glia is also involved in CNS eCB signaling [42]. Both microglia and astrocytes are expressing receptors and enzymes involved in eCB signaling [43,44,45], further shaping the activity of brain circuits through eCBs [46]. Yet, despite the complexity of the ECS and the myriad of unanswered questions, anatomical and functional studies lead to an extensive insight into eCB involvement in physiology and pathology [47]. Prompted by the traditional knowledge that the consumption of THC usually increases appetite even in sated states, it was found that one of the pivotal roles of eCBs is in the control of appetite, feeding and subsequently body weight [48]. In the following sections, we will elucidate the eCB-driven neuromodulation of the underlying brain circuits.

## 2. Endocannabinoids in Central Control of Body Weight

Eating can be seen as the orchestrated output of the nervous system after integrating humoral and neuronal signals balancing energy needs against energy reserves, processing sensory cues, as well as the motivational and emotional state of an individual—constantly weighing in feeding against other survival needs. The classical view distinguishes homeostatic (sustenance-driven) and hedonic (reward-driven) feeding. Simply put, homeostatic feeding will halt once the organism is replete with energy and nutrients, while hedonic feeding might continue. However, all feeding behavior of higher organisms is influenced by brain regions that process reward, centers that integrate aversion versus preference and circuits that make predictions about the future need and availability of food while constantly evaluating hormonal and neuronal feedback from the periphery. Both “hedonic” and “homeostatic” circuitries are intricately interwoven as evidence accumulates that brain regions that have been classically viewed as predominantly involved in homeostatic feeding are influenced by higher corticolimbic and “hedonic” areas of the brain and vice versa [49,50].

In an “obesigenic” environment of easy accessibility and high nutritional density of food, preponderance of the hedonic aspects of feeding without restriction may lead to overeating and obesity [51,52]. To our knowledge, there are no accounts for an obesity epidemic in wildlife, while for humans (as well as for their domestic animals)—in many regions of the world—efforts to obtain calorie-dense, rewarding food are reduced to a minimum and competition for food is virtually absent, and thus, the hard-wired, pro-feeding circuits seemingly promote obesity. One of the important actuators in these circuits and bearers of hope as therapeutic targets are eCBs.

The eCB involvement in body weight control is already shaped early in life: interestingly, in several mammalian species’ milks, including human, 2-AG, the endogenous FAAH-inhibitor oleamide and other eCB-like compounds were found [53]. Furthermore, CB1 seems to be involved in suckling, as blocking CB1 using rimonabant within the first postnatal hours and days of mouse pups prevents milk intake [54]—an effect which is also seen in CB1-knockout (CB1^−/−^) mice on the first postnatal day. However, CB1^−/−^ mice start suckling eventually on postnatal day two or three, suggesting a compensatory mechanism [55]. When mouse pups were orally administered AEA during the nursing period, they exhibited higher body weight, increased fat amount, insulin resistance and higher levels of CB1 expression in adipose tissue in adult life [56,57] as well as altered CB1 signaling in the hypothalamus [58]. However, in this case—as in many studies—central and peripheral effects cannot be clearly distinguished: Is altered hypothalamic eCB signaling the cause or the result of the observed metabolic effects? Infant THC exposure through breastfeeding has been associated with sedation and impaired motor development [59], altered metabolic states have not been described, but it is uncertain whether these effects were considered.

The crucial role of eCBs in the control of body weight has been further demonstrated in global CB1^−/−^ mice, where caloric intake and body weight are significantly lower than in control mice [60] and global CB1^−/−^ mice are resistant to diet-induced obesity (DIO) under a high fat diet (HFD) [61]. On the other hand, globally CB1-deficient mice show significantly reduced life span without any apparent pathology and the cause has not been elucidated [62]. In view of the market withdrawal of Rimonabant as an anti-obesity drug, better knowledge of eCB actions throughout the body are required, especially a separation between central and peripheral effects and a distinction between cause and consequence. Thus, many efforts are still being undertaken to probe eCB signaling in more confined brain regions and organs—in order to understand the underlying mechanisms and provide safe and efficient drug therapies.

### 2.1. Feeling Hungry or Sated: Peripheral Signals and the Hypothalamus

Behaviors associated with feeding often begin with one central feeling that has the power to override all other undertakings of an organism: hunger, an unpleasant feeling of energy need or, complementary, “appetite”, the desire to eat. Initiated by humoral signals such as ghrelin, hypoglycemia and a decline in leptin, activities of neural ensembles throughout the brain prepare the body for one of the most fundamental behavioral patterns: seek food, acquire food, ingest food and digest food. Additionally, numerous autonomous and unconscious processes take place that adjust the body for a state of nutritional deficiency—reduced energy expenditure on the one hand, motivational and sensory focusing towards food intake in anticipation of the rewarding experience on the other hand.

### 2.2. The Hypothalamus Is a Gate for Feeding Behavior

The hypothalamus is considered a center of prime importance in the integration and control of bodily functions essential for survival such as circadian rhythm, body temperature, plasmaosmolarity, as well as feeding. Since a profuse regulation of hypothalamic activity by eCBs has been shown [9,63], we want to put a special emphasis on this circuitry. In order to exert their integrative role in feeding control, hypothalamic neurons show ample expression of receptors for hormones and nutrients and are extensively connected to other brain regions involved in feeding. 

**Figure 2:**
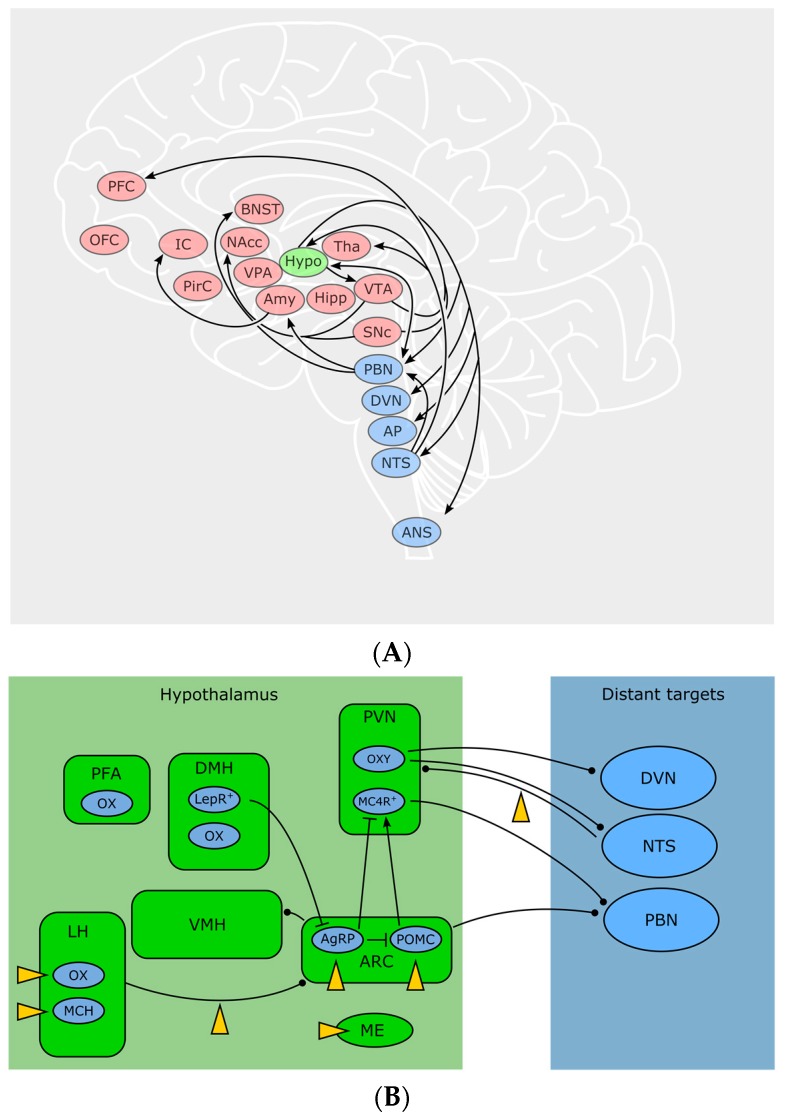
(**A**) Brain regions where the ECS influences different aspects of feeding. Blue: autonomous “hotspots” that convey sensory and visceral information from the periphery to the CNS and vice versa. Green: the hypothalamus is of pivotal significance for the integration of humoral and neuronal signals that evaluate the calorie supply of the whole body. Red: areas especially important for motivation, decision-making, emotion and reward—influencing complex behaviors such as foraging and the choice of food. Abbreviations: PFC prefrontal cortex, OFC orbitofrontal cortex, IC insular cortex, PirC piriform cortex, BNST bed nucleus of the stria terminalis, NAcc nucleus accumbens, VPA ventral pallidum, Amy amygdala, Hypo hypothalamus, Tha thalamus, Hipp hippocampus, VTA ventrotegmental area, SNc substantia nigra, pars compacta, PBN parabrachial nucleus, DVN dorsal nucleus of the vagus nerve, AP area postrema, NTS Nucleus of the solitary tract, ANS autonomic nervous system. (**B**) Pathways and cell types of hypothalamic circuits and their distant connections with the autonomous system. endocannabinoid system (ECS) targets marked by yellow arrowheads. Abbreviations: PFA perifornical area, LH lateral hypothalamus, DMH dorsomedial hypothalamus, VMH ventromedial hypothalamus, PVN paraventricular nucleus, ARC arcuate nucleus, ME median eminence, OX orexin, MCH melanin-concentrating hormone, LEPR leptin receptor, OXY oxytocin, MCR4 melanocortin type 4 receptor, DVN dorsal nucleus of the vagus nerve, NTS nucleus of the solitary tract, PBN parabrachial nucleus.

Hypothalamic neurons occupy a domain especially suitable for sensing blood-borne signals: due to the close proximity to the median eminence (ME), a highly vascularized circumventricular organ lacking the blood-brain barrier (BBB), neurons in this region have direct access to the bloodstream. Anatomically and functionally, one can distinguish more than ten nuclei within the hypothalamus, zoned into an anterior (or “preoptic”), medial (or “tuberal”) and posterior hypothalamus—due to the scope of this review, we will focus on the areas involved in feeding, which are mainly located in the tuberal zone—for a thorough primer on the hypothalamus, see [64]. Basically, a local ECS relevant for body weight control is present in numerous of these specific hypothalamic nuclei. Autonomous, hypothalamic and reward-related feeding areas show a complex pattern of interconnectivity, which remains to be fully disentangled. Some important feeding-related connections discussed here are visualized in Figure 2. For example, the arcuate nucleus (ARC) sends output to other hypothalamic feeding centers: Ventro—and dorsomedial hypothalamus (VMH, DMH), paraventricular nucleus (PVN) and lateral hypothalamus (LH). At the same time, all aforementioned nuclei receive input from the nucleus of the solitary tract (NTS) as well as from the parabrachial nucleus (PBN). Conversely, LH, PBN and NTS are sending output to nucleus accumbens (NAcc) and limbic areas for processes involving reward and motivation, as well as to motor and autonomic areas, for example to the dorsal nucleus of the vagus nerve (DVN). Furthermore, ARC and PVH also send long-range connections to PBN and autonomic motor centers [64].

Located in close proximity to the third ventricle, the ARC contains two reciprocally active neuron populations: ventromedially located Agouti-related peptide (AgRP)/neuropeptide Y (NPY) and dorsolaterally located proopiomelanocortin (POMC) neurons, whose activity codes for hunger and satiety, respectively [65]. ARC neurons assess the caloric need of the body through humoral as well as neuronal signals. Ghrelin activates AgRP/NPY neurons and induces feeding [66]. Leptin depolarizes POMC neurons in the ARC while hyperpolarizing AgRP/NPY neurons [67]. Following fasting, AgRP/NPY neurons are active. During refeeding, a dorsolateral shift of the neuronal activity from AgRP into POMC neurons can be observed, indicating a decrease in hunger and an increase in satiety [65]. POMC and AgRP/NPY neurons innervate the same “satiety” target neurons in the PVN. These neurons express melanocortin receptor 4 (MC4R), a GPCR activated by α-melanocyte stimulating hormone, which is released by POMC neurons, while AgRP is an inverse agonist on these receptors. Additionally, AgRP/NPY neurons inhibit PVN neurons through release of GABA and NPY. The aforementioned MC4R target neurons project to PBN and, when activated, induce satiety behaviors [68]. However, the activity of ARC neurons can be disrupted by cannabinoids and induce a feeding response in a state of satiety with concomitant paradox activity of POMC neurons [48]. Furthermore, presynaptic terminals on AgRP/NPY-neurons (but not the postsynaptic cells themselves) show CB1-expression, suggesting a retrograde control of AgRP activity through eCBs [69].

Besides ARC AgRP/NPY and POMC neurons, other neuronal populations residing in the ARC are involved in feeding control, like dopaminergic neurons [70] as well as orexigenic somatostatin (SST) neurons [71]. As more than 50 transcriptionally different cell types in the ARC-ME complex alone have been identified [71], an even greater complexity of this region, potentially the hypothalamus in general, remains to be unraveled. Moreover, evidence accumulates that ARC cells serve roles beyond feeding, for instance the control of bone mass [72] or immunomodulation through T-cell activation [73] by AgRP/NPY neurons.

Animals with lesioned PVN show increased food intake and obesity [74]. The PVN contains oxytocin-producing neurons that connect to autonomic centers, which eventually send visceroefferents through the vagus nerve [75]. It has been found that oxytocin, besides its well-known functions in bonding, birth and sexuality, shapes vagal parasympathetic output, leading to a decreased food intake [76,77]. Oleoylethanolamide (OEA), a non-CB1 lipid messenger with structural similarities to AEA [78] exerted anorexigenic effects through activation of noradrenergic projections from NTS to PVN and increases oxytocin levels in PVN and supraoptic nucleus [79,80] offering a new pathway connecting the ECS with neuropeptides involved in food intake. 

Integrating peripheral and central signals and information about the environment, the LH orchestrates a broad variety of homeostatic and behavioral functions, such as sleep, stress and anxiety, but also feeding and reward [81]. Cells in the LH are heterogeneous and often classified by the expression of neuropeptides. Prominent representatives are orexin/hypocretin neurons (OX) and melanin-concentrating hormone (MCH) neurons. MCH is increased during fasting [82] and overexpression of MCH leads to hyperphagia and obesity while MCH-knockout causes hypophagia and decreases body weight [83]. Orexin A and B (OX-A, OX-B) were named after their initially observed feeding-stimulating effect [84], and are solely produced in the hypothalamic LH, perifornical area (PFA) and DMH [85]. It was later found that OXs also play an important role in sleep/wakefulness [86] as patients suffering from narcolepsy lack OX expression. Cannabinoids influence the activity of LH neurons in a disparate manner: CB1 activation activates MCH neurons but inhibits OX neurons [87]. Key to this discrepancy might be the organization of the synaptic input to these cells. The innervation of OX neurons depends on the metabolic state of the animal: in lean mice, excitatory input outnumbers inhibitory synapses and the excitatory overbalance even increases after overnight fasting [88]. Expanding on this finding, a study by Cristino et al. [89,90] found that OX neurons in leptin-deficient ob/ob and DIO mice receive predominantly inhibitory input, originating from ARC AgRP/NPY neurons. The driver of this remodeling seems to be impaired leptin signaling in the ARC. At the same time, ob/ob mice show a relative overexpression of DAGLα, leading to higher levels of eCBs and a decrease in inhibitory inputs through DSI. Together with a subsequent study [91], the following mechanism was proposed: OX neurons in wildtype animals use eCB signaling as a negative feedback to dampen excitation. In ob/ob mice however, pathological wiring and enhanced eCB signaling leads to a preferential disinhibition and the relative predominance of excitatory inputs causes a positive feedback that could potentially drive eating behavior despite elevated leptin levels. No similar synaptic rearrangements were seen in MCH neurons [92]. However, in another study, MCH neurons of the LH were also shown to downregulate their inhibitory input through retrograde eCB signaling [93], while leptin—through inhibition of voltage-gated calcium channels—lowers eCB production and subsequently inhibits DSI [93]. This interaction between elevated leptin and eCB signaling could ultimately decrease food intake. Furthermore, CBs also influence the activity in LH target regions. OX-expressing neurons, among other functions, are involved in reward and motivation, as they are active during cues for rewards such as food or drugs and project to reward centers [94]. OX receptors OX1R and OX2R can be found in many brain regions, among them are prefrontal cortex (PFC), ventral tegmental area (VTA), thalamus, hypothalamus, BNST and brainstem [95]. Delivery of OX-A to the hindbrain increases meal size and frequency, potentially through blockade of amylin, a satiety-inducing pancreatic peptide, in the NTS and/or area postrema [96]. A surprising interaction between eCBs and OXs within the hypothalamus has been found by Morello et al. [37]: POMC neurons in the ARC express both CB1 and OX1R and they receive synaptic input by OX neurons of the LH. In obese mice, OX-A signaling was elevated, while POMC and α-melanocyte stimulating hormone transcripts were downregulated in POMC neurons mediated through STAT3. Interestingly, this effect required both OX-A and CB1 signaling, suggesting a potential multi-target pharmacological approach in treating obesity. 

Further evidence for the synergism between OXs and eCBs has been found in projections from LH to VTA, a pathway that might be relevant for the reward-related aspects of food as well: during stress, OX-A release in VTA leads to a 2-AG/CB1-mediated dis-inhibition and subsequently to a reinstatement of cocaine-place-preference in previously extinguished mice [97]. These two examples emphasize the need for studies of feeding regulation that address interactions between neuropeptides and other neuromodulatory systems.

### 2.3. Peripheral Signals Extensively Influence CNS Circuits

In what follows, we will outline the central effects of feeding-related humoral signals. For the discussion of their peripheral actions, the reader is referred to the Section 3. One of the humoral factors evoking hunger is ghrelin, a peptide hormone that is released both in the gastrointestinal tract (mainly in the stomach) and the brain, for example in the hypothalamus [98]. When administered peripherally or centrally, ghrelin stimulates feeding in animals fed ad libitum, but does not further increase acute food intake in fasted or calorie-restricted animals [99]. Midbrain transection abolishes the orexigenic effect of peripherally administered ghrelin [66]. AgRP/NPY neurons are depolarized by the application of ghrelin, both indirectly due to changes in the presynaptic input and directly due to activation of currents in the cell itself [100]. Surprisingly, in CB1^−/−^ mice, ghrelin does not increase feeding [101]. Furthermore, ghrelin application increases eCB levels in the PVN in wild-type, but not in CB1-knockout animals, and this increase can be turned off by CB1-blockade [101]. In addition to the hypothalamus, ghrelin acts through the activation of feeding circuits in the amygdala and the NTS, as well as through the activation of motivation- and reward-related areas such as the dopaminergic projections from VTA to the NAcc [102].

Leptin is a hormone mainly produced in the adipose tissue conveying the status of the energy reserves, acting on medium timescales (hours)—for example, leptin levels remain unchanged within the first 30 min of refeeding after a long fasting and reach control levels after 6 h [65]. While leptin deficiency strongly induces feeding and a decrease in energy expenditure [68], metabolic disorders such as obesity go along with elevated leptin levels but altered responses to leptin, often termed “leptin resistance” [103]. Extra-hypothalamic leptin effects have been found for example in the thalamus during postnatal development [104], in the VTA, where leptin decreases basal and feeding-evoked dopamine, which in turn decreases food intake [105], and in the NTS, where leptin increases pSTAT3 levels without effects on body weight and food intake [106]. It was recently shown that triglycerides cross the BBB and counteract the anorexigenic effects of leptin. Remarkably, leptin uptake in several brain regions was increased upon administration of triglycerides while at the same time leptin- and insulin resistance were observed [107]. The first observation of interactions between leptin and eCBs was made by Di Marzo et al.: Leptin-deficient ob/ob and leptin receptor-defective db/db mice show elevated hypothalamic eCB levels. Administration of leptin to ob/ob mice normalized eCB-levels [108]. When administering the CB1 inverse agonist AM251 and leptin together intraperitoneally (i.p.), food intake and body weight were reduced in rats and this effect was dependent on serotonin signaling. Interestingly, the doses used were subanorectic for each compound individually, showing a synergism between leptin and eCB signaling [109]. Additionally, leptin resistance in DIO mice could be reversed by administration of the peripheral CB1 antagonist JD5037, which surprisingly also decreased hypothalamic AEA levels and hence attenuated central CB signaling, too [110].

Cholecystokinin (CCK) is released by the duodenum during digestion of food. In addition to its effects on the gastrointestinal system, CCK may bind to vagal CCK receptors or CCK receptors 1 and 2 in the brain [111] and induce behaviors associated with satiety. Peripheral injection of CCK is associated with sated behaviors such as halted food intake, less exploration and general inactivity [112]. Following midbrain transections, the behavioral effect of peripherally administered CCK was diminished, as the autonomic neurons in the NTS were disconnected from forebrain structures such as thalamus and hypothalamus, showing that the effects of CCK are not limited to intestinal organs, nor purely autonomic [113]. CCK-expressing neurons in the NTS, as well as another dopamine β-hydroxylase-expressing population, innervate calcitonin gene-related protein expressing PBN neurons. Activation of this pathway leads to decreased food intake and body weight [114]. The CCK and eCB system have been shown to be jointly involved in learning [115] and in circuits involved in anxiety and pain [116]. A feeding-related synaptic connection depending on humoral, neuronal and eCB signaling has been studied by Khlaifia et al.: long term synaptic depression (LTD) between visceroafferent fibers and neurons of the NTS is affected by the feeding state of an animal. Following fasting, elevated ghrelin levels impair eCB-mediated LTD, which can be restored by the elevation of CCK. This mechanism can be thought of as an integrator between visceroafferents and blood-borne signals: the humoral satiety signal CCK attenuates neuronal transmission while the “hunger hormone” ghrelin leads to a more reliable conveyance of afferent signals [117]. Both humoral and neuronal pathways are linked by eCBs, underlining their importance as local modulators, especially in feeding circuits.

Neurons of the ARC and ME express insulin receptors, predominantly axonally located [118]. Insulin decreases NPY expression [119] and hyperpolarizes an insulin receptor expressing subset of POMC neurons [120], contradicting the assumption that—due to the observed anorexigenic effects of intracerebroventricularly (i.c.v.) administered insulin [119]—POMC neurons should be stimulated by insulin [121]. Most interactions between insulin and eCB signaling have been described in the periphery—see Section 3.

Glucagon-like peptide (GLP-1) is secreted postprandially in the gut and was later discovered to be also produced in a subset of neurons of the NTS, innervating hypothalamus (specifically ARC, DMH and PVN), thalamus and cortex [122]. GLP-1 receptors were also found in the BNST, central amygdala and dorsal lateral septum [123]. The identified sites of GLP-1 receptors support the putative role of GLP-1 in decreasing homeostatic and hedonic feeding [124] that have been observed behaviorally [125,126]. OEA and 2-oleoylglycerol have been shown to increase the potency of GLP-1 signaling by binding to GLP-1 directly, suggesting a potential fine-tuning mechanism for this pathway [127]. However, more research investigating the crosstalk between GLP-1 and eCBs and its functional implications, especially in vivo, has to be conducted.

Other than the outlined hormonal signaling systems, several brain regions are also capable of nutrient sensing. For some compounds, the diffusion is facilitated in areas lacking tight junctions of the BBB, for example in the ME as well as in the area postrema.

Fatty acid sensing takes place throughout the brain, is involved in many processes and interwoven with other signaling systems, especially with the ECS [128]. Dietary polyunsaturated fatty acids (PUFA) have been shown to be of importance for processes such as neuroprotection, synaptogenesis and synaptic plasticity [129]. The underlying mechanisms are on the one hand the fact that PUFAs constitute essential components of cell membranes, on the other hand because PUFAs bind to receptors such as GPCR40 and PPAR [129]. Interestingly, a close relationship between PUFA and eCB signaling has been shown. For example, a lifetime dietary deficiency of n-3 PUFAs abrogates CB1-dependent LTD in PFC and NAcc with effects on emotions, namely promotion of anxiety and depression-like behavior in rodents [130].

Protein availability is constantly monitored in the CNS through amino acid sensing. Amino acids cross the BBB through carrier proteins [131]. A substantial body of evidence supports a suggested pathway through which—during a state of deficiency—amino acid sensing neurons in the anterior piriform cortex lead to foraging for a diet that provides essential amino acids required for survival [132]. Furthermore, the i.c.v. application of leucine leads to hypophagic responses mediated by amino acid-sensing centers in the brainstem and hypothalamus [133].

Finally, glucose levels are probably the nutrient signals with the highest priority as severe hypoglycemia is a potentially life-threatening condition. Therefore, neural circuits have emerged that constantly monitor glucose levels and—in case of hypoglycemia—activate the counter-regulatory response through the sympathetic nervous system and increase the likelihood of feeding [134]. Pivotal glucose sensing centers reside in the hypothalamus and brainstem and neuronal glucose sensing has also been found in the peripheral nervous system, for example in the ganglion inferius of the vagus nerve, where almost half of the afferent neurons are either excited or inhibited by glucose [135]. Hypothalamic neurons that sense glucose are POMC and AgRP/NPY neurons of the ARC as well as MCH and OX neurons of the LH [136]. Impairment in glucose sensing mechanisms in POMC neurons, which can be caused by obesity, has been shown to be detrimental for overall regulation of blood glucose levels [137]. Also, glucose sensing is dependent on the metabolic state of the animal [138] and leptin increases glucose sensitivity [139]. Furthermore, glial cells have been shown to be involved in hypothalamic glucose sensing as well: astrocytes sense glucose levels and show altered phenotypes in response to hyperglycemia [140,141] as well as altered glucose uptake following leptin treatment [142]. Interestingly, leptin signaling and glucose sensitivity in astrocytes are linked by the ECS: ablating CB1 in astrocytes interferes with their leptin sensitivity and alters glycogen storage [143]. Moreover, tanycytes are responsive to glucose too [144], their involvement in glucose sensing has been reviewed in [145].

### 2.4. “Wanting” Food: Motivation, Food Seeking and Decision-Making

Food, especially when rich in nutrients and calories, is a primary source of reward [146]—imaginably, as highly nutritious food is usually harder to obtain (e.g., collecting low-calorie plants versus hunting energy-dense game), a rewarding feeling is linked to its consumption and a strong drive to seek for and consume such food served as an evolutionary advantage, especially in respect of human brain development [147]. Reinforcing feelings are already triggered during presentation and anticipation of food intake, which are in combination with food seeking behavior often referred to as the “wanting” aspect of feeding. The “liking” component of feeding relates to the hedonic feelings of pleasure during food consumption [52,148]—often nonspecifically termed “palatability” [149]—and during food digestion. In 1996, Berridge suggested that “liking” and “wanting” are implemented by separable neural circuits and not necessarily conscious [148]. Ultimately, both liking and wanting interact to some extent and are further shaped and by learning processes as most food preferences are acquired and changed throughout life—to an extent that even innately aversive stimuli like bitterness can be overcome due to the link between their consumption and positive feelings, as in coffee, tea and beer [52].

Dopaminergic neurons of the VTA and substantia nigra, pars compacta projecting to a wide array of brain regions are—among other processes—involved in motivation and the incentive value of items and therefore part of the “wanting” system. Interactions between the ECS and dopaminergic circuits are extensive and have been reviewed in [150,151]. I.c.v. injection of ghrelin increases locomotor activity and dopamine release in the VTA, indicating an increased motivation for food seeking. These ghrelin effects can be significantly reduced by i.p. application of the CB1 inverse agonist Rimonabant, while food intake is unchanged [102]. Conversely, leptin decreases dopamine release in the VTA and reduces food intake [105]. Dopaminergic neurons themselves do not express CB1. However, CB1 is present in their GABAergic input terminals which control dopamine release [152]. Accordingly, VTA neurons showed an increased firing rate in response to exposure to synthetic CB1 agonist HU210 in the majority of cells [153].

The NAcc, part of the ventral striatum, is a key recipient of dopaminergic projections from the VTA, also receiving glutamatergic input from PFC, basolateral amygdala (BLA), hippocampus and thalamus [154]. One could think of the NAcc as a system that puts the “wanting” into action in order to achieve “liking” as it integrates diverse inputs and elicits goal-directed behavior [155]. The role of the ECS in the motivational aspects of feeding are beginning to be understood [52] and evidence exists for ECS involvement in many motivation-related areas. For example, fasting induces a strong increase in 2-AG and AEA in the forebrain components of the limbic system [156], indicating eCB modulation of the motivation to acquire food during hunger. Also, experience shapes the activity and organization of the NAcc, partially mediated by eCBs. Low-frequency stimulation of excitatory medial PFC afferents can induce CB1-dependent presynaptic LTD [157], suggesting the possibility that the ECS alters feeding behavior through motivational circuits.

Certainly, our behavior and choices are not exclusively driven by the “wanting” system, as immediate rewards always have to be weighed up against long-term goals of an individual [158]. In order to make choices that are beneficial for the survival of an organism, estimating the value of an item, such as food, is necessary for anticipating the outcome of a certain decision [159]. The orbitofrontal cortex has been suggested to encode specific information about an item and from that, infer anticipated outcomes of a choice, and is therefore, together with the adjacent PFC, involved in decision-making [160,161]. The orbitofrontal cortex encodes both information about the value of an object and value-independent, identity-specific information [159,161]. Identity-unspecific value information however seems to be represented by neurons in the ventromedial PFC in humans. Taken together, parallel circuits are involved in predicting the outcome of a decision [159]. However, in many cases, a decision cannot be easily made, for example when the number of factors to be taken into account exceeds our capacities or when there is a lack of past experience allowing for the estimation of the value of an item. At these times, “wanting” and “liking” may—often subconsciously—help guide our behavior [158]. The underlying behavioral pattern for many eating disorders such as anorexia nervosa or DIO are persistent maladaptive food choices [162]. In a rat model of binge-eating behavior, where female rats had a temporally limited access to HFD in addition to their normal diet, CB1 levels in the PFC were found to decrease in the binge-eating group [163]. Another study showed a slight decrease in PFC AEA levels in mice on HFD compared to standard diet (SD) [61]. Taken together, the ECS in the PFC seems to be downregulated under HFD. When blocking CB1 with low doses of orally administered Rimonabant in rats, food intake was preferentially suppressed for sweet food, while intake of normal chow remained unchanged [164].

The consumption of cannabis sativa has an orexigenic effect on humans, anecdotally especially for highly palatable food. In a study where subjects underwent memory testing, the intake of marshmallows increased significantly after smoking a marihuana cigarette [165]. In addition to this orexigenic effect, THC has been reported to be anorexigenic as well. Oral administration of low doses of THC increased acute food intake in rats, which was compensated by lower food intake afterwards [166], while higher i.p. doses decreased feeding [167]. This is in line with the observation that the feeding response to cannabinoids is “biphasic”, where low doses of THC and AEA have an orexigenic and high doses have an anorexigenic effect [168,169,170,171]. The biphasic feeding response was also seen in sated animals and blocking CB1 with Rimonabant abolished it [171]. Noteworthy, high doses of THC not only decreased feeding but also water intake [169] and an alternative to the explanation that high levels of cannabinoids lead to a feeling of satiety is, that the preponderance of psychotropic and locomotor effects prevents animals from food and water intake (for further discussion, see Section 4. One may speculate whether the increased food intake reflects stronger “wanting” or “liking”. However, evidence exists on THC-increased palatability through the activation of dopamine signaling in the NAcc [172], and by sharpening olfactory sensation [173].

### 2.5. The “Liking” Phase of Feeding: Food Consumption

The perception of taste is essential for the assessment of edibility of food, for the evaluation of its nutritional values as well as—ultimately—the development of food preferences through rewarding experiences and associations [174,175]. Sensory information from taste receptors (see also Section 3) is conveyed to the NTS by the hypoglossal, facial and vagus nerve. From the NTS, taste information in humans is transferred to the PBN of the reticular formation, which is involved in processes such as thermoregulation, arousal and taste and connects to other brain regions related to feeding and reward, such as hypothalamus, thalamus, amygdala and cortex [176]. The infusion of 2-AG to the PBN increases food intake preferably for sweet and fatty food but not for standard chow [177], whereas activation of µ-opioid receptors (MORs)—which show a similar distribution pattern—increased the intake of chow. Furthermore, blocking MOR did not interfere with eCB actions. Therefore, eCBs in PBN seem to constitute a selective reinforcement signal for palatable food [177]. During refeeding following a long fast, there is a significant increase in PBN activity even during the consumption of a standard chow diet [65]. From the gustatory PBN, taste information is conveyed to the NAcc, potentially linking reward to afferent taste information by increasing dopamine levels [178]. Evidence is accumulating that opioid and cannabinoid system are interacting [179]. In addition to dopaminergic control, stimulation of MOR of the NAcc increases the task-dependent consumption of palatable food, which may be caused by enhanced salience of a reward but also due to increased food seeking behavior. Caref et al. found that, when blocking MOR in the NAcc, a decreased cued approach of fatty food is only observed in sated, but not in food-restricted animals, emphasizing the state-dependency of MOR-expressing NAcc neurons promoting food seeking behavior [180]. The NAcc has been shown to express only low levels of CB1 [181] as the major population of cells, medium spiny neurons, which transfer NAcc output to other brain regions, are CB1-negative. Fast-spiking interneurons however, which provide strong inhibitory input to medium spiny neurons, express CB1 in about 40% of the cells. These CB1-expressing fast-spiking interneurons have been shown to become more excitable during cocaine withdrawal [182]. Some eating disorders seemingly share similarities with addictions, such as cravings and over-consumption of food despite knowledge about its negative effects [51]. The NAcc is involved both in reward during addiction as well as food intake. However, whether “food addiction” is a fitting term or whether overeating and binge eating are “just” physiological behaviors taken to an extreme, is debatable [183].

Parts of the “liking” aspects of food intake are processed in cortical areas, like gustatory cortices and insular cortex (IC). The secondary gustatory cortex in primates including humans is located in the orbitofrontal cortex and it connects the primary sensations of smell, taste and texture to reward values [184]. The IC receives visceral inputs through the thalamus as well as through other nuclei in midbrain and hindbrain [184]. In mice, the insular cortex, but not the adjacent somatosensory cortex, is necessary for responding to visual cues that predict food [185]. Livneh et al. found pathways that connect AgRP/NPY neurons to IC through the thalamus and BLA [185]. In the same study, Ca^2+^ imaging insular cortex neurons in wake mice revealed a broad activation pattern during visual cue and food consumption, that did not show any spatial organization. While sated mice did not consume food during presentation of the visual stimulus, chemogenetic activation of hypothalamic AgRP/NPY neurons restored the licking response, potentially mimicking a state of hunger. In the suggested pathway, AgRP/NPY neurons disinhibit BLA neurons through the paraventricular thalamus. BLA sends axon collaterals to IC, putatively providing information about the value of a cued reward [185] and this input might be enhanced during hunger. Support for involvement of the ECS in cortical sensory processing stems from studies in humans suffering from anorexia and bulimia nervosa, where an increased CB1 density in insular cortex and inferior temporal and frontal lobe was found, pointing at a potentially impaired processing of interoceptive, gustatory and reward-related behavior [186].

### 2.6. Digestion of Food: Induction of a Feeling of Satiety

The feeling of “satiety” can stem from different underlying causes—on the one hand from energy replenishment, for example mediated by normalized glucose levels following hypoglycemia, as well as from reaching capacity limits of the digestive tract. While the former could be described as a positive feeling of “replete” as opposed to the latter unpleasant feeling of “stuffed”, we will refer to both processes as “sated”.

The pivotal centers for the control of meal size and meal termination—potential readouts for satiety—lie in the brainstem, controlled by humoral and neuronal afferents from the periphery. In addition to the aforementioned taste pathways relevant during ingestion, visceral afferents from internal organs during digestion are transmitted through the vagus nerve to neurons in the NTS as well as the area postrema via glutamatergic synapses [187]. Located in the medulla oblongata, the NTS connects to forebrain regions as well as to the area postrema, which is a brain region involved in vomiting (see next section), and to the nucleus ambiguus and the dorsal nucleus of the vagal nerve [113]—which influence intestinal motility. Vagal afferents include information from intestinal stretch receptors and gut peptides such as ghrelin [66], glucagon like-peptide 1 (GLP-1), peptide YY and cholecystokinin (CCK), which bind to receptors expressed at intestinal terminals of the vagus nerve [188]; see also Section 3. A deafferentation of the vagus nerve in rats leads to increased meal sizes, which are compensated by lower meal frequency and result in a normal body weight. Furthermore, a nutrient preload of the stomach suppresses feeding just as well as in controls with intact vagal afferents. Taken together, vagal afferents are not solely necessary for the induction of satiety nor maintenance of body weight [189]. 

Brain regions other than hindbrain have been proposed to be involved in satiety-related signaling. In addition to its well-known functions in memory the hippocampus has been shown to be involved in the processing of signals of satiety and regulating appetite [61,190] and hippocampal changes in the ECS related to feeding have been observed: in mice on a HFD, levels of AEA and 2-AG (as well its synthesizing enzyme DAGLα) are significantly increased in hippocampus compared to SD, accompanied by a slight increase in CB1 levels in the stratum radiatum of CA1 and CA3 [61]. Hence, HFD enhances eCB signaling in the hippocampus [61]. Moreover, the observed molecular changes have a functional outcome: upon activation of a cell, DSI was stronger in HFD mice when compared to mice fed normal food [61].

### 2.7. In Case the Food Cannot Be Digested: Nausea and Vomiting 

Nausea and subsequently vomiting are autonomous processes intended to prevent the ingestion or digestion of potentially harmful substances. These feelings can be elicited both peripherally by the GI tract or centrally, in the area postrema [191], triggered by the dorsal vagus complex. Nausea, the uncomfortable feeling that precedes vomiting, as well as vomiting are common side effects of medication and often accompany pathologies [192]. Especially in cancer, these side effects of chemotherapeutics can aggravate tumor-associated weight loss severely and hence are important symptoms to treat. Cannabis sativa has been known for its antiemetic properties for a long time [193]. However, careful examination of the underlying processes is essential, as chronic cannabis consumption lead to frequent vomiting for reasons yet unknown [194]. ECS influences on nausea and vomiting have been reviewed in [191], potential therapeutic interventions will be discussed below.

### 2.8. Expanding the Neurocentric View: Glia in Feeding Control

In addition to a neurocentric view on feeding circuits, glial cells recently drew increasing interest as they have been shown to participate in feeding control. Astrocytes, the most abundant type of glia in the CNS, show versatile phenotypes across brain regions and a tendency to adapt to anatomical and physiological properties of their surrounding neurons [195,196,197]. Due to astrocyte ability to shape synaptic transmission and neuronal activity [47,198,199,200] by forming close interactions with synapses (termed “tripartite synapse” [201]) and through the release of “gliotransmitters” [202,203], one can imagine that astrocytes are involved in feeding control.

Yang et al. reported that astrocytes are capable of reducing food intake through the increase in extracellular adenosine, whose A_2A_ receptor has been shown to form heteromers with CB1 [92,204]. Astrocytic adenosine release in the hypothalamus inhibits the activity of AgRP/NPY neurons of the ARC both basally as well as following ghrelin stimulation [205]. On top of this, astrocytes have been shown to be critically involved in glucose-mediated effects in the hypothalamus. The cell-specific knockout of their insulin receptors impairs glucose uptake to the CNS and leads to altered metabolism and behavior in response to glucose elevation [206,207]. The fact that hypothalamic astrocytes are important elements in hypothalamic feeding circuitry is further supported by the findings that, during postnatal development, astrocytes proliferate in response to leptin [208] and the knockout of astrocytic leptin receptor blunts leptin-induced feeding suppression and induces hyperphagia [209,210]. Providing a cellular basis for astrocyte involvement in eCB signaling, Navarrete et al. showed that astrocytes express CB1 and respond to eCBs released by neighboring pyramidal cells [43]. Another study found that astrocytic eCB sensitivity can mediate heterosynaptic long-term-potentiation (LTP) through the release of gliotransmitters, suggesting a glia-dependent pathway by which eCB signaling can affect synapses located remotely from the eCB release site [46].

Tanycytes are a specialized type of radial glia surrounding the third ventricle, making contact with both the portal capillaries and the cerebrospinal fluid. Generally, tanycytes express a broad variety of receptors for neuropeptides important for the hypothalamic feeding circuitry and distinct types of tanycytes can be distinguished—for a review on tanycytes, see [211]. Tanycytes have for example been shown to be involved in glucosensing [144,145], amino acid sensing [212] and leptin sensing [213]—in the latter study, tanycytes were shown to exert abnormal functions in leptin transport in ob/ob and DIO mice, emphasizing their critical role in a circuit that was mainly studied with a neurocentric view so far. Tanycytes show polar DAGLα-immunostaining [214], ordering investigation of tanycyte-produced eCBs and their effects on the nearby feeding circuitry [215].

Microglia are the resident macrophages in CNS parenchyma. As HFD causes an inflammatory response in the brain [216], a proliferation of microglia can be observed [217]. Blocking this microglial proliferation ameliorates HFD-induced pathologies such as adiposity and leptin resistance [217]. Moreover, microglial activation has been shown to modify neuronal activity in feeding circuits: inflammatory activation of ARC microglia changes synaptic input to and altered activity of POMC neurons, leading to a sickness behavior in mice [218]. Similarly to eCB-mediated immunomodulation in the periphery, microglia phenotypes can be altered by eCB activation as well [45], suggesting an additional pathway through which the eCB imbalance in hypothalamic feeding circuits alters neuronal activity. Buckley et al. revealed through CB2-knockout studies that the immunomodulatory effects in peripheral tissues are mediated by CB2 [219], but found that binding of a synthetic agonist was unaffected in the brain, supporting the prevalent role of CB1 in the CNS. However, it was later shown that microglia, as the principal immune cells of the CNS, do express CB2 [220,221] with functional implications both in health [222,223] and disease [224]. However, microglial expression of CB2, which is upregulated during microglial activation [225], remains difficult to visualize and quantify as basal expressions seem low and detection methods are unreliable [226]. Noteworthy, CB2 signaling was not only shown to exert an anti-inflammatory role, it also affected cognitive processes such as contextual fear memory, shown in a study by Li and Kim [223]. For further discussion of the immunomodulatory effects of eCBs see section ‘The emerging role of the hepatic and pancreatic ECS in metabolic disorders’.

The evidence for glial involvement in homeostatic and feeding circuits as well as in eCB signaling, it becomes clear that research investigating the eCB involvement in body weight control should span all cell types as they potentially provide the “missing link” for the multitude of unexplained eCB effects. 

### 2.9. Back from the Brain to the Periphery: Neuronal Output Influencing Metabolism

In the previous section, we reviewed how signals from the periphery influence the CNS and how this information is integrated and processed in a variety of circuits. As mentioned earlier, global CB1^−/−^ mice do not develop an obese phenotype when fed a HFD [61]. Interestingly, following CB1-knockout specifically in GABAergic neurons, body weight on SD is equal to control mice, but on HFD, visceral fat and body weight over time are lower. As the calorie intake is equal to control animals, these GABAergic cells may be involved in a circuit that regulates energy expenditure rather than food intake [61]. Similarly, in a study by Quarta et al., the anorexic and weight decreasing effects of rimonabant were ablated in mice with a CB1-knockout directed to glutamatergic, calmodulin-dependent protein kinase-expressing cells [227]. It was shown that these mice exhibit an overactivity of the sympathetic nervous system and increased thermogenesis, mediated by a pathway from forebrain to NTS and from there to the periphery, leading to an improved metabolic profile. In what follows, we want to examine the evidence for peripheral effects of eCB signaling and the consequences for body weight control.

## 3. Endocannabinoids in Peripheral Body Weight Control

### 3.1. Peripheral eCB Signaling in Metabolic Health and Disease

From a plethora of investigations on metabolism and body weight control, it emerged that the ECS is not only a partaker in the aforementioned brain circuitries but also represents an elementary factor in numerous peripheral organs in control of energy metabolism and consequentially in the regulation of body weight. In this chapter, we will focus on the indispensable role of the ECS for the regulation of food digestion, nutrient transformation and energy expenditure due to the interactions between eCBs and signaling cascades in the gastrointestinal (GI) tract, liver, pancreas, fat depots and endocrine glands.

Basically, all compounds of the ECS described before are also present in the body’s periphery. Both CB1 and CB2 show strong expression in peripheral tissues. For example, CB1 is robustly detectable in liver hepatocytes, adipocytes of white fat depots, as well as in different cell types of the GI tract, pancreas and skeletal muscles. In contrast, CB2 is predominantly expressed in immune and blood cells, where eCBs mediate immunomodulatory actions. Besides the well-established contribution of the ECS in regulation of energy metabolism in the body’s periphery under physiological conditions, the overall involvement of eCBs in modulation of inflammatory events [228,229] also accounts for pathophysiological processes in metabolic diseases, such DIO or type 2 diabetes [230]. The first evidence that eCBs are important for body weight regulation via peripheral CB1 activation came from a study by Cota et al. in 2003 [231]. The lean phenotype of CB1^−/−^ mice under normal chow feeding and the resistance against DIO, accompanied by maintenance of insulin sensitivity after high fat feeding, suggested that eCB signaling in DIO not only leads to hypothalamic alterations, but also to peripheral impairments in the liver, pancreas and adipocyte tissue [231]. In connection to these findings, induction of lipogenesis in adipocytes by peripheral CB1 activation was described [231] and hepatic CB1 was shown to be responsible for development of diet-induced steatosis, dyslipidemia, insulin- and leptin resistance [232,233,234]. In this regard, it was found that the main degrading enzymes for eCBs such as AEA and 2-AG show very high expression levels in the adipose tissue and the liver [235,236]. Moreover, the levels of eCBs in these peripheral organs depend on the nutrition state. For example, induction of DIO alters the activity of the enzymes for the synthesis and degradation of AEA and 2-AG [23,230,237,238,239,240].

**Figure 3:**
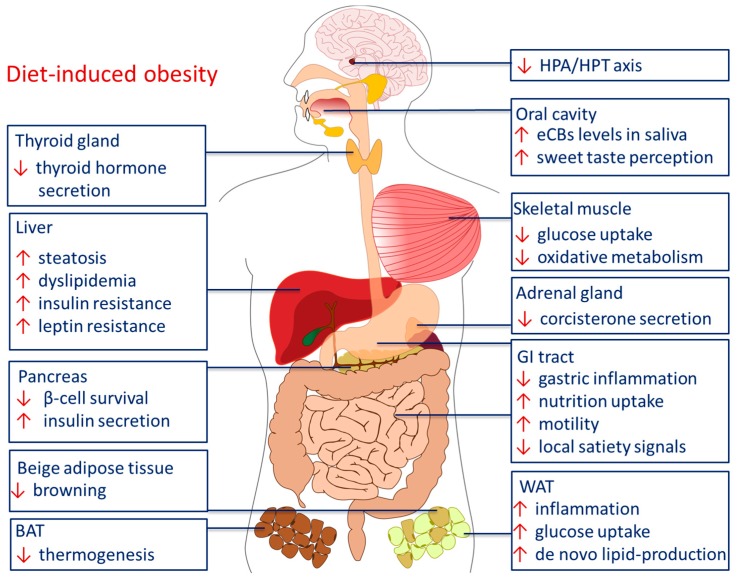
Peripheral effects of the ECS in diet-induced obesity. Abbreviations: BAT brown adipose tissue, HPA hypothalamic pituitary adrenal axis, HPT hypothalamic pituitary thyroid axis, GI gastrointestinal, WAT white adipose tissue.

These observations were accompanied by several studies in mice and rats showing that chronic treatment with rimonabant reduces body weight, independent of central regulation of food intake [241,242,243,244]. Moreover, detrimental parameters in the course of DIO, such as increased levels of blood glucose and triglycerols, as well as hyperinsulinemia and -leptinemia were reversed after treatment with CB1 inverse agonists [23,233]. Due to these findings, one major approach in obesity research is to focus on selective peripheral inverse agonists and neutral antagonists to treat obesity, in order to avoid central psychotropic side effects. This aspect will be outlined in the final chapter of this review. An overview of the peripheral effects of the ECS in DIO is shown in Figure 3.

In the upcoming sections, we will follow cannabinoid effects throughout the sequence of food intake and digestion, starting at the very first stage in the mouth, where the eCBs affect taste sensation [245,246,247,248] and influence secretion of saliva by modulation of the vegetative innervation of the salivary glands [249]. We will then follow the way of the ingested nutrients through the GI tract, and describe the role of the eCBs in the communication between the GI tract, liver, pancreas, skeletal muscles, fat depots and the brain. Finally, we will also address the role of the ECS in the neuroendocrine axes between hypothalamus, pituitary, adrenal and thyroid glands.

### 3.2. The ECS in The Oral Cavity: Taste Sensation and Saliva Production

Increased levels of eCBs were observed in the saliva of obese, insulin resistant individuals during fasting compared to fasting normal weight individuals [249]. Therefore, salivary eCBs might serve as biomarkers for obesity. Another group confirmed these findings and found that besides the increased levels of eCBs in the saliva of obese subjects, also the levels of uric acid and C-reactive protein were upregulated [250].

The content and composition of dietary fat plays a crucial role for the perception of taste. Fat as a primary taste quality is detected by several receptors in the oral cavity. An innate attraction to fat-rich nutrition might be due to the oro-sensory detection of dietary lipids leading to dopamine outflow in the ventral striatum. In this region of the midbrain, sensory stimuli are processed and “wanting” and “liking” (see Section 2) lead to a stimulation of increased intake of fat [251].

Experimentally, rodents showed a strong preference for diets rich in fat and containing linoleic acid. This preference is lost in knockout-mice lacking the CD36 fat receptor, which is expressed for example in taste buds [246,247]. Binding of a long-chain fatty acid at the CD36-receptor causes a signaling cascade that induces the release of neuromediators and gastrointestinal hormones [252]. The sensory information is conveyed to the NTS via the gustatory nerves and transmitted through a reflex loop via the vagus nerve to the peripheral axis where an early secretion of digestive enzymes and hormones [253,254] takes places in order to prepare the body for incoming lipids. Another study showed that the free acid component of dietary fat advanced the accumulation of eCBs in the proximal small intestine. Oleic acid and linoleic acid are the respective components of the diet, which triggered this effect [251]. The authors suggested that the fat-sensing effect mediated by CD36 and lingual lipase activity are involved in the initiation of eCB signaling in the jejunum, but this interaction must be investigated in more detail in further studies [251].

“The fatter the food, the more palatable” holds true for humans as well, and therefore, dietary fat influences our eating habits. Therefore, it is not surprising that in the “western diet” the percentage of linoleic acid raised from 1% to 8% of total energy intake in the last century, strongly contributing to the raise in obesity due to ingestion of energy dense foods [255]. Most strikingly, the content of our diet, especially the percentage of fat, and the kind of fat can influence the levels of eCBs in brain, small intestine and liver [256].

Sweet taste perception is also influenced by eCBs [245,257]. The study of Yoshida et al. showed a significantly increased activity of the chorda tympani, which innervates the anterior tongue, triggered by sweet compounds after i.p. injection of 2-AG and AEA [245]. This effect was observed 10–30 min after injection in wild type mice and was diminished to control levels 60–120 min after injection. Moreover, this effect was absent in CB1^−/−^ mice [245]. This increase was only observed in response to sweet compounds, as 2-AG showed no effect on the nerve response to the other tastes qualities salty, bitter, sour or umami [245]. Moreover, many single nucleotide polymorphisms (SNPs) exist at the gene locus of the sweet receptor, leading to a high variability and consequently influence taste perception and personal food preference [174,258].

However, the perception of sweet taste is more complex due to the fact that there are many factors involved in this sensation, because paracrine and endocrine hormones influence the sensibility of the taste receptor and consequently modify the palatability of food and eating behavior. The different players in this modulation process are leptin, CCK, NPY, oxytocin, insulin, ghrelin and galanin [259]. Yoshida et al. suggest an interaction between leptin and the ECS in regard to sweet taste sensitivity, which may influence eating behavior and energy homeostasis via central and peripheral mechanisms [174,248,260]. In leptin receptor-defective (*db*/*db*) mice, the nerve response to sweet sensation is decreased when blocking CB1 [248]. To sum up, the ECS is of significant relevance in the oral cavity by modulation of taste sensation of sugar and fat. Finally, saliva production itself is influenced by the ECS [261,262].

### 3.3. The ECS in the GI Tract

All key players of the ECS are present in the stomach and intestine, allowing for the local de-novo synthesis of AEA, 2-AG and OEA. These eCBs operate in an auto-, para- and endocrine fashion. When activated by eCBs, CB1 in the GI tract induces GI motility, reduces the secretion of acid and fluid and accelerates mesenteric vasodilation [263]. Furthermore, the activated ECS mediates a reducing effect on gastric damage and intestinal inflammation [264,265]. These essential anti-inflammatory aspects of the ECS optimize the uptake of the nutrients in the GI tract. Besides CB1 and CB2, other eCB receptors exist in the GI tract, namely TRPV1 [266], the PPAR class [267], as well as GPR119 [268] and GPR55 [269]. For example, OEA, an eCB-like compound generated on demand from enterocytes, is an agonist at PPARα [270], TRPV1 channels [271] and the orphan GPCRs GPR55 and GPR119 [272], but not to CB1 and CB2 [273]. The production of OEA is induced by food intake [274] and repressed by food deprivation [275]. OEA mediates satiety by PPARα activation [276], decreases food intake and consecutively body weight gain [277]. PPARα is a nuclear receptor influencing various aspects of lipid metabolism [270]. Fu et al. suggest that satiety, induced by PPARα activation, is mediated by the reduction of nitric oxide (NO) through transcriptional downregulation of the intestinal nitric oxide synthase [270,278]. OEA also triggers the release of the anorexigenic hormone GLP-1 by binding to GPR119 of enteroendocrine L-cells [279,280].

The stomach and small intestine send humoral signals to the brain to control of energy balance, and different studies suggest that the ECS is involved in these pathways [281]. In this, the peripheral ECS in the GI tract strongly affects the secretion of classical humoral factors like ghrelin, CCK and GLP-1 [282]. The central dopamine deficiency caused by a HFD is probably linked by intestinal OEA levels [283]. Supplementation of OEA under HFD leads to restoration of dopamine levels accompanied by the intake of less palatable food with reduced fat content [283], illustrating the potential of gut-derived eCB to affect central reward circuit systems. 

Conversely, the type of food which we consume, can, for its constitution of different components, influence the ECS. The study of Monteleone et al. demonstrated an increase of the 2-AG and ghrelin plasma levels of healthy volunteers when they are allowed to consume their favorite food which was mostly high in fat and sugar [284]. In experiments with rodents that had access to a diet rich in fat and sucrose, both features of the “Western diet”, heightened levels of AEA and 2-AG in plasma and additionally in the jejunum were observed, accompanied with a greater portion intake and weight gain [285]. Additionally, our high-fat western diet has a high ratio of n-6/n-3 PUFAs, today the ratio is 20:1 or even higher in comparison to evolution in which the ratio was 1:1 [255]. It is thus assumed that this drastic increase goes along with the development of obesity. The ratio of n-6/n-3 PUFAs has an impact on the AEA [286,287] and 2-AG levels [288]. In an interventional study by Berge et al., obese men were supplemented with krill powder, which contains the n-3 PUFAs docosahexaenoic and eicosapentaenoic acids. This supplementation resulted in a decrease of AEA and triglyceride levels in plasma and to a reduction of ectopic fat accumulation [286]. Accordingly, animal studies showed that a diet rich in n-6 PUFA and poor in n-3 PUFA increases brain levels of AEA [287] and of 2-AG [288]. Similarly to other phospholipid-derived compounds [289,290], the underlying mechanism might be an altered availability of substrates for eCB synthesizing enzymes. 

Another route through which the ECS interacts in the regulation of food intake are vagal afferents, which link the GI tract to the medulla and brainstem nuclei related to satiety to supervise the process of food digestion. After food intake, the duodenum secretes CCK, which then binds to CCK receptors located at afferent terminals of the vagus nerve [108]. The incoming signal is then transferred to the hypothalamus to reduce food intake. It was shown that upon CB1 activation, the release of CCK in the duodenum is inhibited, presumably involving enteroendocrine L-cells expressing gene transcripts for CB1 [291].

Besides these effects, eCBs also seem to interact with intestinal microbiota. The first indication that there exists a communication between the ECS and the gut microbiota was reported in 2007 [292]. The presence of the bacterial strain *Lactobacillus acidophilus* induces the expression of cannabinoid receptors and MOR in intestinal cells lowering abdominal pain in rats. Also, THC consumption affects the gut microbiome [293]—for further discussion, see the Section 4 of this review.

Together, all these findings represent an effect of diet on the ECS in the intestinal tract suggesting an association between western-style diet and the involvement of eCB signaling in hyperphagia [251,294].

### 3.4. Liver

Under physiological conditions, the hepatic ECS is assumed to be idle. If it reaches a pathophysiological state, as in the course of DIO, the ECS will be activated [23]. Such a pathophysiological state is for example hepatic steatosis, induced by HFD or excessive alcohol consumption. Hepatic steatosis is linked to an upregulation of liver CB1, triggered by retinoic acid, which is produced by hepatic stellate cells [295]. When activated CB1 was chronically blocked with an inverse agonist like rimonabant, the process of steatosis could be reversed [233,296].

CB1^−/−^ mice show a resistance to the development DIO and to the development of the accompanying hepatic steatosis. HFD induces lipogenesis in the hepatocytes [232]. Activation of hepatic CB1 increases lipogenesis and concomitantly inhibits fatty acid oxidation, the catabolic process of lipid metabolism [233,234]. The ECS-promoted accumulation of fat in the liver depends on ATP. There is a negative correlation between the ATP content of the liver and the insulin resistance of hepatocytes and CB1 in the liver serves as an important modulator of hepatic energy status [295]. Another study showed a decreased FAAH activity in hepatic steatosis, presumably causing a rise in eCBs, especially in AEA. Monounsaturated fatty acids, which are produced by the enzyme stearoyl CoA desaturase-1, lead to a depression of FAAH activity. Stearoyl CoA desaturase-1 is an enzyme in the liver whose expression is induced upon HFD [297]. Other enzymes and proteins involved in fatty acid synthesis and consecutively the development of hepatic steatosis are upregulated by eCB-driven activation of hepatic CB1: sterol regulatory element binding transcription factor 1, fatty acid synthase and acetyl coenzyme-A carboxylase-1 [232].

A physiological key feature of the liver is the production of bile acids, which are required for absorption of ingested fats. It was shown that hepatic CB1 contributes to alcohol-induced shifts in the expression of bile acid metabolizing enzymes, involving ER-bound transcription factor Crebh (cAMP-responsive element binding protein, hepatocyte specific) as downstream CB1 effector [298]. Interestingly, the activity of NAPE-PLD, one of the key enzymes involved in biosynthesis of *N*-acetylethanolamines such as AEA, OEA and PEA, is controlled by bile acids [299,300]. Here, the binding of bile acids enhances dimer assembly of NAPE-PLD, which is required for catalytic activity. Since the various products of NAPE-PLD carry different effects on feeding and energy metabolism, for example, AEA can be orexigenic, while OEA serves as an important satiety signal, it would be interesting to see whether there exists a physiological interrelation between the ECS and bile acid composition and under which circumstances this putative interaction accounts for body weight control in healthy normal weight and obese people.

### 3.5. Pancreas

The pancreas secretes digestive enzymes into the duodenum. In cultured lobules and acini of guinea pig and rat pancreas, both CB1 and CB2 involvement in exocrine secretion was observed [301]. In an experimental model of acute pancreatitis, it was shown that CB2 signaling led to reduction in inflammation via MAPK signaling, finally affecting cytokine release [302]. Thus, it would be interesting to test for the functional contribution of the ECS in the exocrine part of the pancreas under normal and HFD.

Compared to the exocrine pancreas, the ECS is well known for its contribution to blood glucose control, mostly by direct interference with endocrine cell types of the pancreatic Langerhans islets [230,303]. Different studies revealed an impact of ECS on the control of β-cell function [304,305]. However, differing opinions still exist regarding which cell type in the Langerhans islets expresses which type of cannabinoid receptor. While there are studies showing that CB1 mRNA and protein are expressed in α-cells and CB2 is expressed in both α- and β-cells [239,306,307], most studies agree on the presence of eCBs and expression of CB1 in β-cells and an increase of insulin release leading to activated CB1 receptors [239,308,309].

Pancreatic β-cells influence themselves via an autocrine anti-apoptotic feedback loop: insulin binds to the insulin receptor and positively regulates the survival of β-cells. Kim et al. performed in vitro studies demonstrating that the phosphorylation of the pro-apoptotic protein B-cell lymphoma 2 (Bcl-2)-antagonist of cell death is reduced, leading to the inhibition of the insulin receptor kinase activity. The hypothesized underlying mechanism is the formation of a heteromeric complex between CB1 and the insulin receptor [310]. Also, TRPV1 was found in both α- and β-cells of mouse pancreatic islets. It was supposed that this receptor is involved in the development of the pancreas as its genetic knockout or pharmacological blockade results in an increased ratio of β- to α-cells, which finally causes an increased islet size [311].

### 3.6. The Emerging Role of the Hepatic and Pancreatic ECS in Metabolic Disorders 

Obesity-associated inflammation in liver, pancreas and white adipose tissue (WAT), accompanied by insulin resistance and hepatic steatosis, was potentiated by pharmacological CB2 receptor activation and diminished in globally CB2-deficient mice. This suggests a selective CB2 antagonism in DIO as a potential pharmacological strategy to exert metabolic benefits [228]. However, and in contrast to that, numerous data also point toward a beneficial role of CB2 activation in metabolic control in lean as well as in obese and diabetic rodent models [230]. For instance, it was demonstrated that CB2 activation improved glucose tolerance in lean rats, supporting that CB2 is relevant for physiological control of glucose metabolism [312]. In the same study, both CB1 and CB2 were observed in rat pancreatic β- and non-β-cells, illustrating putative interactions between CB1 and CB2 in glucose homeostasis [312]. While CB1 activation contributes to body weight gain and onset of metabolic syndrome, CB2 signaling is thought to mediate contrariwise beneficial effects, aiming at anti-inflammation and reversal of metabolic syndrome [313]. Indeed, CB2 was described to increase protective effects in a model of diabetic nephropathy [229]. Altogether, once the mechanisms underlying the inflammation in metabolic organs are understood, the impaired fat metabolism in the adipose tissue could be overcome by the development of pharmaceuticals, which can treat the inflammation and repress it to a baseline level.

### 3.7. Skeletal Muscle

Following digestion and uptake, nutrients are utilized by muscle cells, which, as an important metabolic entity, make use of eCB signaling [314,315,316,317,318,319]. Activation of the ECS decreases insulin-stimulated glucose uptake and oxidative metabolism in human skeletal muscle [314,315]. The decrease in oxidative metabolism is caused by inhibition of substrate oxidation and inhibition of mitochondrial biogenesis, similarly to results obtained in liver and adipose tissue [320]. The negative correlation between the activated ECS and insulin is triggered by an impact of CB1 on the PI 3-kinase/PKB and on the Raf-MEK1/2-ERK1/2 pathways [319]. Activation of the TRPV1 channels in the skeletal muscle stimulates mitochondrial biogenesis and hypertrophy [321,322]. However, there is no evidence that eCBs are involved in these TRPV1-mediated processes in the skeletal muscle.

A study by Crespillo et al. demonstrated that the activity of the skeletal muscle ECS depends on the consumed diet. Treatment with an inverse CB1 agonist restores HFD-induced alterations in skeletal muscle cells [316], highlighting the importance of the ECS in muscle cells for metabolic health and body weight control.

### 3.8. Adipose Tissue

Besides WAT, the ECS is also present in brown adipose tissue (BAT), where it contributes to the thermogenic function of fat cells and regulates body weight by directly affecting energy expenditure [237]. In WAT, eCBs and leptin are negatively correlated [323]. In times the ECS is stimulated in adipose tissue, cascades for energy storage are activated, leading to increased de novo production of lipids and glucose uptake. As a consequence, the expression of the hormone adiponectin, a cytokine with anti-inflammatory features, is downregulated, which has an impact on the insulin sensitivity at distant tissues like the skeletal muscle and the adipose tissue itself, and, on top of that, causes a local inflammatory process in the adipose tissue [324,325].

Obesity and its comorbidities are often accompanied by inflammation of the adipose tissue, which is suggested to accelerate the onset of metabolic syndrome [326]. Therefore, many groups aim at the identification of new pharmaceutical targets to influence this adipose tissue inflammation [327,328]. While specific targets are upregulated in the course of DIO-associated inflammation of WAT, such as the transcription factor E2F1 [329], it is overall accepted that inflammation in metabolically active organs links the development of insulin resistance and liver diseases to pathways of the immune system [330,331]. In DIO, the treatment with Rimonabant reverses the downregulation of adiponectin, causing anti-inflammatory effects [324]. Analysis of human subcutaneous adipose tissue of obese participants, when compared to lean controls, revealed a decrease in FAAH activity, increased eCB levels but a decreased expression level of the CB1 receptor [238]. The authors suggest that CB1 may be regulated by a negative feedback loop and that its downregulation is a secondary effect of the increased eCB levels. In this regard, the same study showed an upregulation of CB1 and FAAH in mature human adipocytes in contrast to pre-adipocytes, highlighting the physiological relevance of the ECS in mature human adipocytes [238]. As previously mentioned, CB1 activation results in adipogenesis and lipogenesis, which leads to an impaired mitochondrial function in DIO [231,332,333]. Upon CB1 activation, there is a downregulation of PPARγ coactivator 1a (Ppargc1a), triggering a decrease of mitochondrial mass and function in WAT. In contrast, a blockade of CB1 the expression of Ppargc1a is increased leading to an elevated mitochondrial biogenesis [320,333].

Activated CB1 favors WAT and inhibits thermogenesis in BAT and beige adipose tissue [334]. The effect on BAT is presumably mediated by CB1-induced inhibition of the sympathetic tone. Accordingly, the pharmacological blockade of CB1 results in differentiation of white into beige adipocytes [335]. Similar to brown adipocytes, beige adipocytes have an enriched number of mitochondria and a higher activity of the enzyme AMPK and uncoupling protein 1 (UCP1). In addition to their common task in thermogenesis, beige and brown adipocytes show many distinguished characteristics, as beige adipocytes are derived from another embryonic precursor cell [336]. Within subcutaneous WAT, clusters of beige adipocytes, can develop due to different stimuli [337]. 

Normally, the BAT protects our body against cold environments using high-caloric nutrients for the required energy [338]. A cold environment leads to noradrenaline release from sympathetic neurons which activates lipolysis in BAT and WAT through activation of β3-adrenoceptors [237,339]. In BAT, the released fatty acids are transferred to mitochondria for β-oxidation and heat production, depending on the presence of UCP1. UCP1 enables the exothermic production of ATP, resulting in heat production, required for stabilization of the body temperature [339]. In BAT, the ECS represents an autocrine negative feedback mechanism: after cold exposure, β3-adrenoceptor activation increases eCB levels in BAT, which in turn attenuate the sympathetic tone and thereby decrease browning [237]. Overall, specific targeting of adipocyte CB1 represents an interesting interventional approach in order to treat obesity and metabolic syndrome [340], since a recent study clearly indicated that adipocyte CB1 plays a key regulatory role in the crosstalk among adipocytes, immune cells, and the sympathetic nervous system [341].

### 3.9. The ECS in Neuroendocrine Circuitries Being Relevant for Body Weight Control

The pituitary gland is an important endocrine interface between the hypothalamus and the peripheral endocrine glands. With regard to body weight control, the hypothalamic-pituitary-adrenal (HPA) and hypothalamic-pituitary-thyroid (HPT) axes represent the most significant functional systems and will be addressed here.

### 3.10. Hypothalamic-Pituitary-Adrenal Axis (HPA) and the ECS

The activation of the HPA axis due to stress is necessary for survival. This axis is regulated by different brain structures and is adjusted by eCB signaling [168,342]. In this regard, there clearly exist site-specific roles of the ECS within the HPA, and divergent functions of AEA and 2-AG in the HPA were observed [343]. While a few studies report on acute activation of the HPA triggered by cannabis consumption or by the use of CB1 agonists, numerous studies revealed that the ECS is involved in stabilization of the HPA axis under physiological, basal conditions, while upon stressful mediators, the ECS is thought to dampen the stress response finally allowing for the recovery of homeostasis [344].

Indeed, CB1 was detected in both the pituitary and adrenal gland [345,346]. The authors showed that CB1 is located in human adrenal cortex cells and that peripheral steroidogenesis and cortisol release are inhibited by synthetic cannabinoids [347]. Further studies demonstrated that inhibition of the ECS results in an increase of circulating corticosterone concentrations in animal models of stress, like forced swimming and tail suspension [348,349]. Injection of CB1 and CB2 inverse agonists into the third ventricle acutely increased serum corticosterone levels in stressed rats [344]. In accordance to these results, previous studies demonstrated that i.p. AM251 treatment raised both, the basal control and stress-induced levels of HPA-axis activity [350,351,352]. In line with this, elevation of eCBs, as induced by treatment with FAAH inhibitor, decreased the stress-induced corticosterone serum levels [344]. However, another study showed that acute central application of AEA induced secretion of adrenocorticotropin (ACTH) hormone [353]. The release of ACTH is not influenced by the genetic deletion of CB1 or the pharmacological treatment with CB1 blockers [354]. The authors suggested that there is another interaction between the ECS and the HPA axis besides the pituitary. Indeed, the adrenal gland expresses CB1 but not CB2 in the cortex [347]. ACTH, as a main regulator of steroid biosynthesis and -secretion, promotes corticosterone secretion, which is inhibited by AEA. Interestingly, this effect was only partially reversed by CB1 blockers, but completely reversed by the blockade of TRPV1 [344]. These findings link AEA, as a full agonist of TRPV1 channels, with peripheral effects of ACTH on adrenal cortex, since ACTH induces expression of the TRPV1 channel [355].

### 3.11. The ECS in Hypothalamic-Pituitary-Thyroid (HPT) and Growth Hormone (GH) Axes

Besides important developmental functions such as control of CNS maturation and longitudinal body growth, the thyroid hormones thyroxine (T4) and triiodothyronine (T3) represent indispensible regulators of thermogenesis and energy metabolism and therefore contribute to body weight regulation [356]. In this, hypothalamic thyrotropin-releasing hormone (TRH) stimulates the synthesis and secretion of pituitary thyrotropin (TSH) in the anterior lobe, which finally activates biosynthesis and secretion of T4 and T3 in the thyroid gland. Moreover, a close functional interrelation exists between the HPT and GH axes [357]. In the anterior pituitary, GH release is stimulated by the hypothalamic GHRH (growth hormone-releasing hormone) [358]. The application of CB1 agonists let to decreased GH levels [359]. This effect was presumably mediated indirectly by cannabinoid-dependent inhibition of GHRH release in the hypothalamus [358]. Thyroid hormones and GH interact in regulation of insulin-like growth factor-1 (IGF-1) levels [360]. IGF-1 represents an important metabolic molecule, which induces cell growth and differentiation. In this regard, the ECS is suggested to be involved in growth control by regulation of the GH/IGF-1 axis [361].

Administration of AEA led to an acute decrease in TSH and T4, but not T3 levels in rat serum, while application of CB1 inverse agonists acutely raised TSH levels [362]. These findings are overall in line with previous reports showing that THC reduces serum TSH, T4 and T3 [363], while the synthetic CB1/CB2 agonist WIN 55212-2 reduced T4 and T3, but did not alter TSH levels [364]. In this regard, central CB1 in the hypothalamus are thought to mediate the acute effects of cannabinoids on the HPT axis. Basically, glutamatergic synapses that contain CB1 and contact with TRH neurons in the PVN were identified [365]. Accordingly, studies showed that THC application reduced TRH amounts [366], and eCBs directly affected activity of TRH positive parvocellular neurons in the PVN, potentially by eCBs driven DSE at glutamatergic input synapses [367]. Thus, a general negative modulation of the HPT axis by the ECS is postulated. Besides these central effects, eCBs might also be able to directly modulate TSH and T4/T3 secretion since CB1 was detected in pituitary and thyroid gland as well [368].

## 4. Therapeutic Targeting of the ECS in Body Weight Regulation—Clinical Implications and Pharmacological Perspectives

### 4.1. The Medical Potential of the ECS in Treatment of Pathological Weight Loss

Historically, both, the recreational consumption of marihuana and the controlled administration of THC promoted appetite and resulted in increased food intake in sated humans [369]. However, a hyperphagic response to THC was not observed in all human individuals [370]. Specifically, THC consumption led to favored intake of caloric dense palatable foods, accompanied with moderate weight gain upon chronic application within a few weeks [371,372]. It was also observed in these studies that by smoking marihuana, the orexigenic effect of THC was more pronounced at lower when compared to higher doses, assuming a biphasic feeding response upon THC treatment [369]. In mice, when cannabinoids were applied in a sub-psychotropic dose range, lower doses of e.g., THC increased while higher (still sub-psychotropic) doses decreased feeding [369,373]. Besides stimulation of food intake, CB1-driven decrease of energy expenditure may also account for treatment of body weight loss. Indeed, CB1 activation at postganglionic sympathetic neurons induced sympatholytic effects finally resulting in reduced energy expenditure [227]. Together, pharmacological activation of CB1 induces appetite, promotes food consumption and reduces energy expenditure and thus might be helpful for patients suffering from chronic loss of appetite and severe reduction in body weight.

Thus, numerous indications exist that pharmacological promotion of CB1 signaling will potentially result in body weight regain in patients suffering from anorexia. Anorectic patients suffering from psychiatric disorders such as anorexia nervosa or bulimia, or patients affected by the cancer anorexia-cachexia syndrome could potentially benefit from CB1-activation [9]. Unfortunately, it is still controversially discussed whether treating anorexia with CB1 agonists represents a promising therapeutic option or not. Many clinicians are worried about the acute and chronic psychotropic effects of CB1 agonists, and the state of scientific knowledge on use of cannabinoids in the clinics in order to treat anorexia is still enigmatic [374,375]. While the overall possibility of decreasing body weight by pharmacological blockade of CB1 signaling is evident, a fully established and well-accepted pharmacotherapy which increases food intake and results in body weight gain by promoting CB1 signaling is still lacking.

One reason might be the fact that the orexigenic effect of CB1 agonists strongly depend on the individual’s metabolic state, since the orexigenic effect was most pronounced in sated persons when compared to fasted individuals [370]. As reviewed above, this orexigenic effect of cannabinoids is primarily associated with central CB1 signaling leading to increased food seeking, amplified sensory detection of food, consumption of caloric dense palatable food and downregulation of energy expenditure. Notably, chronic consumption of marihuana, e.g., for recreational purposes, does not necessarily lead to development of severe obesity or metabolic syndrome, potentially due to pharmacological habituation. Another assumption in this regard was that chronic consumption of THC might lead to shifts in the gut microbiome upon prolonged treatment, which finally also may account for limitations in cannabinoid-driven weight gain [293].

In the group of patients suffering from the cancer anorexia-cachexia syndrome, tumor by-products and/or host cytokine release combined with metabolic abnormalities might lead to an imbalanced ECS in both, central and peripheral circuitries [376,377]. While THC safely and effectively induced caloric intake, mood and sleep in anorectic HIV patients [378,379], orexigenic effects of THC were not detected in a phase III study on patients with cancer anorexia-cachexia [380]. This still equivocal set of clinical studies might be due to the lack of resilient data obtained in phase I/II studies carefully eliciting pharmacokinetic, dose-concentration and concentration-response data in cancer patients suffering from anorexia [381]. Principally, it is suggested that a chronically underactive ECS exists under anorectic conditions [186,377]. In patients suffering from anorexia or bulimia nervosa, an upregulation of CB1 was observed in cortical and subcortical areas of the brain [186]. It is supposed that under anorectic conditions, eCB-driven pathways contribute to abnormal hedonic input into brain areas managing sensory, interoceptive and motivational signals. Major differences for CB1 density in anorexia and bulimia nervosa patients were observed in the insular cortex, an area that not only codes for sensory detection of taste, flavor and oral texture of food, but also for rewarding properties of food [382,383]. Alongside altered CB1 density was detected in frontal and temporal areas of the cortex, regions well known for their interoceptive abilities required for integration of a variety of different sensations [384]. However, the main question remaining open here still is whether the variations observed for CB1 in anorexia reflect a cause or consequence of the disease. Nevertheless, only a few clinical trials have already taken place which have shown that the treatment with dronabinol, a dual CB1/CB2 agonist, leads to a little but significant weight gain in anorexia rodent models and humans with anorexia nervosa, potentially due to a reduction in the urge to be physically active [376,377]. In line with these findings, dronabinol reduced activity and attenuated weight loss in a rat model for activity-based anorexia [375]. Many cancer patients are suffering from nausea and vomiting in the course or after chemotherapy. In this, CB1 agonists displayed a well-tolerated anti-emetic drug, reducing nausea and vomiting, however, besides these acute beneficial effects, only some short-term improvement in appetite was detected, with no or some long-term improvements in body weight were documented in these patients [376].

In conclusion, much more research is needed to clarify the pathological role of the ECS in anorexia. Moreover, the pharmacokinetics of selective CB1 and CB2 agonists or of dual CB1/CB2 agonists have to be tested more systematically in clinical trials aiming at therapy of anorexia [381]. Alongside, another pharmacological approach in order to induce CB1/CB2 signaling could be based on inhibitors blocking specific enzymes responsible for degradation of eCBs [385]. In mouse models for anxiety or pain, pharmacological or genetic blockade of eCB degrading enzymes such as FAAH or MAGL led to increased levels of AEA and 2-AG, respectively, which finally resulted in analgesic and anxiolytic effects [386,387,388]. However, the pain-reducing effects observed in mouse and rat models have not yet been successfully transferred into humans [389]. In order to treat depression (anxiety)-like behaviors, in which FAAH blockade was described as a successful therapeutic option in mouse models [390], it recently occurred that participants in a phase 1 study of a compound known as BIA 10-2474, a presumed selective FAAH inhibitor, were hospitalized with severe neurological symptoms, presumably due to off-target proteins [391]. Overall, this tragedy finally indicates that ongoing further research on the ECS is still required and that much more critical considerations are needed for the performance of clinical trials that are based on interspecies translational approaches when regarding the ECS. Fortunately, a collaborative effort between multiple academic and industry laboratories revealed that HU910, HU308 and JWH133 represent the most selective CB2 agonists and thus being the most recommendable candidates to test for CB2 selective effects in pathophysiology of anorexia [392]. Basically, neuroinflammatory alterations are associated with neuropsychiatric disorders and polymorphisms in the CB2 gene have been reported not only in depression and schizophrenia but also in eating disorders [393]. Thus, besides CB1, the selective targeting of CB2 might be also relevant for the pharmacological treatment of eating disorders. Indeed, a polymorphism of the CB2 gene could be associated with anorexia nervosa and bulimia [394]. In mouse models, CB2 blockade by AM630 decreased food intake under non-fasting conditions while the same drug (AM630), when administered following food deprivation, increased food intake [395,396]. Thus, while antagonism of the CB1 receptor induces anorexia irrespective of fed or fasted states, the effects of CB2 receptor agonists on food intake appear to depend on the current metabolic state. This finally indicates that both CB1 and CB2 affect food intake in rodents, although the underlying mechanisms remain to be determined [393].

### 4.2. The Medical Potential of the ECS in Treatment of Overeating and Obesity

As highlighted above, chronic activation of the ECS is strongly linked with obesity and its co-morbidities. For example; plasma eCBs are not only elevated in obese patients but also in patients with type-2 diabetes [397]. In this disease the dietary intake of fatty acids plays an important role in determining tissue eCB levels [287,288]. Thus, CB1 blockade was thought to represent a useful tool for the treatment of obesity. In 2006 the first generation of CB1 inverse agonists represented by rimonabant and other “nabant” drugs was discovered in Europe [398]. Rimonabant, marketed as Acomplia (in Europe) and trademarked as Zimulti (USPTO, Washington, DC, USA) was the first clinical approved CB1-dependent drug. Its main properties represent restoring insulin sensitivity in DIO, normalizing fat cell size, preventing visceral fat accumulation and decreasing subcutaneous fat [399,400,401]. Acomplia also improved cardiovascular risk factors such as low adiponectin, high HDL and high triglyceride levels [402]. The underlying mechanisms, however, were largely unknown. In this regard, the first attention has focused on adiponectin [403]. Plasma adiponectin as well as the adiponectin gene expression in visceral fat was increased during rimonabant treatment. Also expression of adiponectin receptor 1 and 2 was enhanced; hence it is proposed that the increase of the adiponectin gene expression elevated the adiponectin delivery into the liver [404]. Furthermore, rimonabant plays a liver-protecting role in obesity by reducing inflammatory reactions and increasing fat oxidation, resulting in a decreased accumulation of lipids in the liver [405]. Additional studies showed that peripheral but not central injection of rimonabant elicited decreased triglycerides in WAT, illustrating that fat reduction induced by rimonabant is independent on its central effects on food intake [406]. In this, rimonabant further induced activation of the sympathetic nervous system highlighting that bidirectional circuits between the periphery and the brain are involved in CB1-dependent regulation of feeding [407]. Unfortunately, rimonabant not just reduced body weight independently of reduction in food intake but also resulted in high levels of psychiatric side effects [408]. Due to this devastating effect, Acomplia was pulled from markets in Europe, never received a FDA approval in the US and research focus has shifted to sole peripheral CB1 inverse agonists to eliminate the CNS side effects. Today, rimonabant is thus considered as an unacceptable treatment of obesity and its co-morbidities [398], and one of the most relevant pitfalls for research associated with the relevance of body weight control was the clinical failure of rimonabant [409].

### 4.3. Omitting Central CB1—Is It Sufficient to Medicate Morbid Body Weight Solely by Selective Targeting of Peripheral CB1?

Several other first generation “nabant” like inverse agonists have failed phase 2 or 3 clinical trials due to the aforementioned undesirable CNS effects described for rimonabant [244]. Thus, aiming at avoidance of undesired psychotropic side effects, blood-brain-barrier impermeable CB1 inverse agonists, such as JD5037 or TM38837 and global neutral antagonists that are brain penetrant, such as AM4113 were developed as second generation CB1-dependent pharmaceuticals, and so far have been successfully tested in rodent models of obesity and metabolic syndrome [403]. Since several studies suggested that the central side effects of Rimonabant were due to its structure as an inverse agonist, brain penetrant neutral CB1 antagonists might avoid these detrimental CNS effects. Indeed, this assumption holds true for the anti-obesity effects of AM4113, a drug, which although reaching the brain did not show typical central side effects as induced by “nabant” like inverse agonists before [410]. Moreover, the use of the neutral antagonists at putative lower doses will account for less central side effects as well [403]. Overall, second generation CB1 blocker showed the same beneficial metabolic effects when compared to rimonabant, but instead did not show detrimental central side effects in animal models. Administration of global or peripheral neutral antagonists, as well as of peripherally restricted inverse agonists showed great beneficial potential for the treatment of obesity and metabolic disease. Here the peripherally restricted CB1 inverse agonist JD5037 and the neutral antagonist AM6545 have to be mentioned. Both of them reduced obesity, reversed leptin resistance and improved hepatic steatosis, dyslipidemia and insulin resistance [403,411]. Moreover, peripheral blockade of CB1 led to the recovery of central leptin sensitivity [110,412]. There are still ongoing studies designing and testing for other new generation CB1 blockers, such as “Compound 2p” and “Compound 10q” which peripherally target the ECS, and look very promising as an alternative treatment of metabolic diseases [403]. In this regard, putative milestones for targeted drug discovery presumably will be the discovery and description of the crystal structure of human CB1, as revealed in complex with AM6538 and taranabant as stabilizing antagonists, respectively [413,414]. Moreover, crystal structure of agonist-bound human CB1 showed important conformational changes in the overall structure in relation to the aforementioned antagonist-bound state [415]. Altogether, the recent discovery of the CB1 crystal structure should lead to the design of chemically diverse ligands with distinct pharmacological properties [415].

As discussed before for anorexia, pharmacological targeting of CB2 might also have therapeutic implications to treat overeating and obesity. While CB1 is increased in obese rodents, CB2 is decreased in peripheral tissues, arguing that CB2 possibly opposes the pro-obesity effects of CB1 signaling [416]. Indeed, CB2 is present in metabolically active tissues, such as liver, pancreatic islets, adipose tissue and skeletal muscle [417]. Besides its localization, the inhibition and/or deletion of CB2 led to an increased food intake in non-obese rodents as well as increased body weight and adipose tissue hypertrophy [417]. Thus, recent studies discuss the possibility of CB2 stimulation in order to reduce food intake and body weight gain without having an impact on mood [416,417]. Chronic treatment of DIO mice with the CB2 agonist JWH015 reduced food intake and fat mass of retroperitoneal and inguinal WAT as well as adipocyte cell size [417]. In more detail, body weight loss was accompanied by increased markers of lipolysis, elevated expression of the anti-inflammatory cytokine IL-10 and by reduction of the pro-inflammatory marker TNF-alpha [417]. Thus it appears that by silencing the activated immune system, which has a key role in worsening obesity and metabolic diseases, CB2 signaling might obtain anti-obesity effect. This assumption is further supported by the findings that age-associated obesity was pronounced in CB2-deficient mice fed a normal laboratory chow [416].

### 4.4. Positive and Negative Allosteric CB1 Ligands: New Therapeutic Avenues for Treating Eating Disorders and Restoration of Morbid Body Weight?

*In silico* mapping of allosteric binding sites at human CB1 supports the idea that body weight regulating effects being transduced by orthosteric CB1 ligands could potentially be affected by allosteric ligands [418]. Numerous synthetic and natural allosteric CB1 modulators with negative or positive effects on orthosteric ligand (cannabinoid) binding efficacy were described so far in vitro [419]. Several endogenous small molecules such as lipoxin A4, pregnenolone and PEPCAN-12 were identified as intrinsic allosteric CB1 ligands [419,420]. Being part of the Hpa (a-hemoglobin-derived peptide hemopressin: PVNFKLSH) neuropeptide family, PEPCAN (peptide endocannabinoid)-12, also known as RVD-hemopressin, was first described in 2012 as a negative allosteric modulator of CB1 [421]. Interestingly PEPCAN-12 is a non-lipid molecule being released from noradrenergic neurons in the CNS, and was shown to decrease food intake in obese mice [420,422]. Another structurally similar peptide called hemopressin is also considered as an allosteric CB1 inhibitor. When compared to CB1 inverse agonists, hemopressin also shows a dose-dependent hypotensive effect in mice [423,424]. It further decreased food intake in normal and obese rodents without any adverse side effects [424]. However, many allosteric ligands so far being described and tested in vitro have not yet shown a sustained effect on CB1 signaling in vivo [425]. Thus, research still has to put a lot of effort into the successful establishment of allosteric CB1 ligands as potential future pharmacological tools in the clinics. Before, much more insight into mechanistic properties of allosteric CB1 ligands is required. For example, it was shown that ORG27569, PSNCBAM-1, and PEPCAN-12 decreased eCB-driven DSE in autoptic hippocampal neurons [422]. Using the same experimental setup, positive allosteric modulators of eCB-driven effects in neurons were identified as well [426]. Finally, another natural allosteric CB1 inhibitor is represented by the neurosteroid pregnenolone. It was shown that pregnenolone binds to CB1 without affecting binding of orthosteric agonists. Downstream effects of pregnenolone are thought to be independent of adenylyl cyclase/cAMP-driven pathways, but should occur via inhibition of the MAPK pathway. Chronic administration of pregnenolone does not cause anxiety in DIO mice [422].

In conclusion, research on allosteric mechanisms at CB1 and other target sites for eCBs is of great interest and high relevance in basic research. Here, the overall goal should be the generation of mechanistic insights in order to develop safe and reliable drugs being able to treat morbid body weight regulation in underweight, overweight and obese patients worldwide.

Overall, the knowledge about the involvement of the ECS in body weight control increased significantly in the last years and is still growing. Recent mechanistic insights into eCB-driven pathways participating in body weight control, and the design of novel pharmacological tools might lead to a major breakthrough in the development of cannabinoid medicines for treatment of adverse body weight development.
